# *In vivo* and *In vitro* Interactions between *Pseudomonas aeruginosa* and *Staphylococcus* spp.

**DOI:** 10.3389/fcimb.2017.00106

**Published:** 2017-04-03

**Authors:** An Hotterbeekx, Samir Kumar-Singh, Herman Goossens, Surbhi Malhotra-Kumar

**Affiliations:** ^1^Laboratory of Medical Microbiology, Vaccine and Infectious Disease Institute, University of AntwerpWilrijk, Belgium; ^2^Molecular Pathology Group, Cell Biology and Histology, University of AntwerpWilrijk, Belgium

**Keywords:** microbial interactions, *S. aureus*, quorum-sensing, cystic fibrosis, biofilm

## Abstract

The significance of polymicrobial infections is increasingly being recognized especially in a biofilm context wherein multiple bacterial species—including both potential pathogens and members of the commensal flora—communicate, cooperate, and compete with each other. Two important bacterial pathogens that have developed a complex network of evasion, counter-inhibition, and subjugation in their battle for space and nutrients are *Pseudomonas aeruginosa* and *Staphylococcus aureus*. Their strain- and environment-specific interactions, for instance in the cystic fibrosis lung or in wound infections, show severe competition that is generally linked to worse patient outcomes. For instance, the extracellular factors secreted by *P. aeruginosa* have been shown to subjugate *S. aureus* to persist as small colony variants (SCVs). On the other hand, data also exist where *S. aureus* inhibits biofilm formation by *P. aeruginosa* but also protects the pathogen by inhibiting its phagocytosis. Interestingly, such interspecies interactions differ between the planktonic and biofilm phenotype, with the extracellular matrix components of the latter likely being a key, and largely underexplored, influence. This review attempts to understand the complex relationship between *P. aeruginosa* and *Staphylococcus* spp., focusing on *S. aureus*, that not only is interesting from the bacterial evolution point of view, but also has important consequences for our understanding of the disease pathogenesis for better patient management.

## Introduction

Over the past decade there is a growing appreciation that the biofilm mode of growth is the most common lifestyle adopted by bacteria (Hall-Stoodley et al., 2004; Burmolle et al., 2014). Biofilms can be defined as surface-associated, structured bacterial communities embedded in an extracellular matrix (Hall-Stoodley et al., 2004). Living in a biofilm provides protection in a stressful environment where mechanical stress, desiccation, and biocides are common threats (Donlan and Costerton, 2002; Flemming and Wingender, 2010). Multiple species frequently exist together in a single biofilm, where they either improve the fitness of one another or compete for space and nutrients (Jefferson, 2004; Billings et al., 2013; Burmolle et al., 2014; DeLeon et al., 2014). Most bacteria have developed interaction strategies to communicate within and between species in a cell density-dependent manner, for example, by using small diffusible molecules in a process called quorum-sensing (Federle and Bassler, 2003; Li and Tian, 2012). Furthermore, many bacteria excrete antimicrobial components, also often regulated by quorum-sensing, to eliminate competitors (Federle and Bassler, 2003; Li and Tian, 2012). Indeed, these multispecies interactions within the biofilm are important for the inhabiting bacteria and, given the increasing evidence of the link between biofilm-associated pathogens and disease, also from a clinical point of view (Donlan and Costerton, 2002; Li and Tian, 2012).

*S. aureus* and *P. aeruginosa* are important pathogens causing a wide variety of infections, including pneumonia in cystic fibrosis (CF) patients, healthcare associated pneumonia and chronic wounds (Harrison, 2007; Fazli et al., 2009; Cystic Fibrosis Foundation Patient Registry, 2012). Initially, only an antagonistic relationship between both organisms was described as the presence of one is associated with the absence of the other in CF and both are rarely found in close association in chronic wounds. *S. aureus* mostly resides on the wound surface whereas *P. aeruginosa* is found in the deep layers (Kirketerp-Moller et al., 2008; Fazli et al., 2009). We also recently showed a negative correlation between presence of *P. aeruginosa* and the total species diversity in *in vivo* endotracheal tube biofilms and a low co-occurrence of *P. aeruginosa* with *Staphylococcus epidermidis* (Hotterbeekx et al., 2016). Nonetheless, recent studies have also co-isolated *P. aeruginosa* and Gram-positive bacteria, including *S. aureus*, from the same infection site where increased virulence and/or antibiotic resistance is described (Duan et al., 2003; Kirketerp-Moller et al., 2008; Fazli et al., 2009; Dalton et al., 2011; Korgaonkar et al., 2013). After describing first *in vivo* observations occurring in human diseases, we will discuss and summarize *in vitro* data from the current literature on potential mechanisms of interactions between *P. aeruginosa* and *Staphylococcus* spp., primarily *S. aureus*.

## Co-occurrence of *P. aeruginosa* and *S. aureus in vivo* is linked to worse disease outcomes

CF is a typical example of a biofilm-related infection wherein *P. aeruginosa* and *S. aureus* are frequently isolated from the lungs of these patients (Harrison, 2007; Hauser et al., 2011; Cystic Fibrosis Foundation, 2014). While, *S. aureus* is mostly acquired during childhood, the presence of *P. aeruginosa* is associated with increasing age and worsening patient prognosis (Sagel et al., 2009; Hauser et al., 2011; Cystic Fibrosis Foundation, 2014). An increasing incidence of *P. aeruginosa* with age has been shown to coincide with a decreasing *S. aureus* incidence in CF patients (Harrison, 2007; Cystic Fibrosis Foundation, 2014), data that primarily indicates an antagonistic relationship between the two pathogens. However, in cases where *P. aeruginosa* and *S. aureus* have been co-isolated, both pathogens seem to contribute independently and additively to the disease severity (Sagel et al., 2009; Hauser et al., 2011), presenting as increased lung inflammation and consequently increased lung damage compared to infection with a single pathogen (Sagel et al., 2009). Furthermore, due to repeated antibiotic therapy, CF patients also carry higher levels of methicillin-resistant *S. aureus* (MRSA) that is associated with a worse lung function compared to methicillin- sensitive *S. aureus* (MSSA) but only in combination with *P. aeruginosa* (Hubert et al., 2013).

Chronic wounds are another example of biofilm-related infections wherein co-presence of *P. aeruginosa* and *S. aureus* has been shown to result in delayed wound healing compared to single species infections (Dalton et al., 2011; Seth et al., 2012; Pastar et al., 2013). In a pig wound model, infections initiated by *in vitro* preformed dual species biofilm caused a significant suppression of keratinocyte growth factor 1 (KGF1), which is responsible for re-epithelialization and wound closure (Pastar et al., 2013). In a rabbit ear-wound model, mixed species infection of *S. aureus* and *P. aeruginosa* caused an increased expression of the pro-inflammatory cytokines IL-1β and TNF-α, indicating a higher inflammatory response compared to single species infection (Seth et al., 2012). Moreover, *S. aureus* and *P. aeruginosa* reached an equilibrium after 12 days of infection, with *P. aeruginosa* being the dominant pathogen (Seth et al., 2012). In a mouse chronic wound model infected with *in vitro* preformed four-species biofilm and monitored up to 12 days, presence of multiple species was found to significantly delay wound healing only at 8 days post-infection (Dalton et al., 2011). However, polymicrobial infections showed increased antimicrobial tolerance compared to single species infection with *P. aeruginosa* in this study (Dalton et al., 2011). These studies suggest that, despite the constraints of different host backgrounds, multispecies infections can lead to delayed wound healing, increased inflammation and increased antibiotic tolerance, which all add to a worse patient outcome. *P. aeruginosa* is often the dominant pathogen due to its wide array of mechanisms to adapt to changing hostile environments, which allows colonization in a variety of niches. When *P. aeruginosa* encounters other bacteria like *S. aureus*, it can co-exist or take over the biofilm through production of various quorum-sensing regulated factors. Section Extracellular products of *Staphylococcus* spp. impact *P. aeruginosa* virulence *in vivo* discusses *in vivo* animal studies exploring production of *P. aeruginosa* virulence factors in the presence of *S. aureus*.

## Extracellular products of *Staphylococcus* spp. impact *P. aeruginosa* virulence *in vivo*

**Figure 1:**
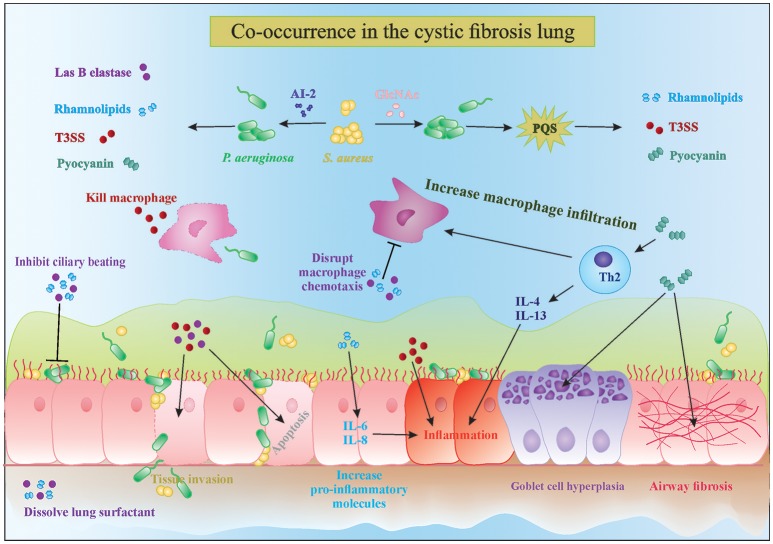
**Interactions in the cystic fibrosis lung**. The complex interplay between microbial competition and the human immune system results in increased secretion of pro-inflammatory cytokines, microbial virulence factors and consequently tissue damage and bacterial invasion of the epithelial barrier. Mixed species infections lead to a stronger decrease in ciliary beating and increased goblet cell hyperplasia and tissue fibrosis, which are characteristic of cystic fibrosis disease progression. PQS, Pseudomonas quinolone signal; T3SS, type 3 secretion system; AI-2, autoinducer 2.

*P. aeruginosa* possesses a wide range of extracellular factors to survive and invade human tissues, often by modulating the immune system. The complex interplay between biofilms and the host immune response are reviewed in detail by Watters et al. (2016). Here, we discuss four molecules in particular which are upregulated in the presence of Gram-positive bacteria: LasB elastase, rhamnolipids, exotoxins, and phenazines (Figure 1). LasB elastase is an extracellular protease capable of digesting the lung surfactant, the pulmonary antimicrobial enzyme lysozyme, and transferrin, as well as slowing down the ciliary movement (Hauser, 2009). In addition, LasB impairs uptake of *P. aeruginosa* by macrophages and its protease activity leads to lung tissue damage, thereby decreasing pulmonary function and facilitating dissemination into the bloodstream (Strateva and Mitov, 2011). Similar to LasB, rhamnolipids are glycolipidic biosurfactants that interfere with the lung surfactant activity by solubilizing the phospholipids and with airway immune response by disrupting the polymorphonuclear leucocyte chemotaxis and macrophage function, and also inhibit ciliary beating (Soberon-Chavez et al., 2005; Jensen et al., 2007). Furthermore, rhamnolipids increase inflammation by stimulating the release of the pro-inflammatory cytokines IL-6 and IL-8 by the airway epithelium (Soberon-Chavez et al., 2005). An excessive inflammatory response and associated tissue damage is also induced by the release of exotoxins ExoT, ExoS, and ExoY by the type III secretion system. The type III secretion system is a needle-like structure directly injecting exotoxins into other bacteria, macrophages and epithelial cells, thereby killing them (Hauser, 2009). The fourth type of molecules, phenazines, are pigments produced by a large number of *Pseudomonas* spp., and have been shown to be involved in mediating microbial interactions as well as in CF disease progression. Pyocyanin is the most important phenazine and its production in the CF lung was shown to lead to goblet cell hyperplasia, airway fibrosis and alveolar airspace destruction (Caldwell et al., 2009; Strateva and Mitov, 2011). Pyocyanin causes an imbalance between the T helper type 1 (Th1) and type 2 (Th2) cytokines, leading to overproduction of Th2 cytokines IL-4 and IL-13 and increased macrophage infiltration (Caldwell et al., 2009). Essentially, release of these extracellular molecules by *P. aeruginosa*, partly in response to the presence of *S. aureus*, leads to increased tissue damage due to their cytotoxic and immune-modulatory effects, which also helps *P. aeruginosa* survival. Interestingly, not only *S. aureus* but Gram-positive commensals (coagulase negative staphylococci and viridans streptococci) can also alter the virulence of *P. aeruginosa* in a similar fashion (Duan et al., 2003). Such immune modulation and evasion by collective bacterial species might underlie the worsened patient prognosis observed in multispecies infections (Figure 1). Sections AI-2 in the CF Lung Increases *P. aeruginosa* Virulence and Might Be an Important Therapeutic Target and N-Acetyl Glucosamine Sensing Enhances the Production of *P. aeruginosa* Extracellular Virulence Factors discuss the known mechanisms of how extracellular products of staphylococci modulate the four major virulence factors *P. aeruginosa* that were discussed above.

### AI-2 in the CF lung increases *P. aeruginosa* virulence and might be an important therapeutic target

Autoinducer-2 (AI-2) is a small diffusible quorum-sensing molecule produced by several bacteria, including staphylococci, and has been shown to cause upregulation of several major virulence genes of *P. aeruginosa* discussed above, including extracellular protease (*lasB*), rhamnosyltransferase involved in rhamnolipid synthesis (*rhlA*), exotoxins (*exoT, exoS, exoY*) and phenazines (*phzA1* and *phzA2*; Li et al., 2015). Induction of *P. aeruginosa* virulence by AI-2 was shown both *in vitro* after screening of a random *lux* reporter-based promotor library and *in vivo* in rat lung infection and *Drosophila* chronic infection models (Duan et al., 2003; Sibley et al., 2008; Li et al., 2015). AI-2 mediated quorum-sensing is now recognized as a universal language of interspecies communication regulating a wide variety of genes involved in virulence and biofilm formation in a cell-density dependent manner in a number of micro-organisms, including non-producers like *P. aeruginosa* (Rezzonico et al., 2012). Furthermore, AI-2 has been detected in substantial amounts in the sputum of CF patients and in infected rats (Duan et al., 2003), raising the possibility of interruption of AI-2 signaling to either slow down disease progression or hasten the healing process.

A promising approach is the use of AI-2 analogs like D-ribose that block the AI-2 pathway and inhibit *P. aeruginosa* virulence. Wang et al showed in a rat model of mechanical ventilation that co-inoculation of *P. aeruginosa* and *Streptococcus mitis* resulted in increased biomass, lung damage, and rat mortality compared to infection with only *P. aeruginosa* (Wang et al., 2016). Treatment with D-ribose of both the single and dual species infections showed a significant decrease in biomass and lowering of rat mortality in the latter group due to interference with AI-2 signaling (Wang et al., 2016). Inhibition of *P. aeruginosa* virulence is not only beneficial because the *P. aeruginosa*-mediated damage is reduced but also because the immune system is less stimulated (Figure 1). Further studies showing the benefits of non-toxic biofilm inhibitors such as D-ribose in patient populations are awaited.

### N-acetyl glucosamine sensing enhances the production of *P. aeruginosa* extracellular virulence factors

**Figure 2:**
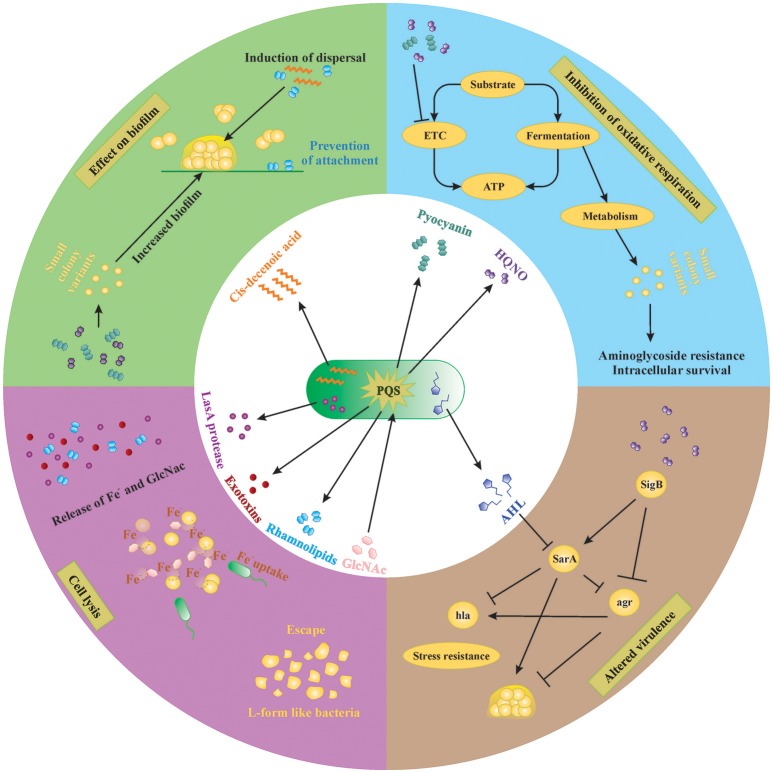
**Small molecules secreted by ***P. aeruginosa*** and ***S. aureus*****. Extracellular factors produced by *P. aeruginosa* affect biofilm formation, oxidative respiration, cell lysis and virulence of *S. aureus*. Lysis of *S. aureus* leads to increased extracellular iron and N-acetyl glucosamine (GlcNac), which are sensed by *P. aeruginosa*. AHL, N-acyl homoserine lactone; HQNO, 4-hydroxy-2-heptylquinoline N-oxide; PQS, Pseudomonas quinolone signal; GlcNac, N-acetyl glucosamine.

Another molecule that increases the virulence of *P. aeruginosa* and is commonly found in the CF lung is N-acetyl glucosamine (GlcNAc). GlcNAc is part of the Gram-positive cell wall polymer peptidoglycan and induces the virulence of *P. aeruginosa* by enhancing the *Pseudomonas* quinolone signal (PQS), which controls the production of extracellular virulence factors like pyocyanin, elastase, rhamnolipids and HQNO (discussed in Section Pseudomonas Quinolone Signal Regulates the Production of Anti-Staphylococcal 4-Hydroxy-2-Heptylquinoline N-Oxide (HQNO); Deziel et al., 2004; Williams and Camara, 2009; Jimenez et al., 2012; Korgaonkar et al., 2013; Figures 1, 2). The PQS, with 2-heptyl-3-hydroxy-4-quilonone as the main effector molecule, is one of the three quorum-sensing systems present in *P. aeruginosa*. PQS is positively regulated by LasR and negatively regulated by RhlR, the two other quorum-sensing systems of *P. aeruginosa* with N-acylhomoserine lactone as main effector molecule (Jimenez et al., 2012). *P. aeruginosa* has the ability to sense the peptidoglycan shed by the Gram positive commensal flora and in response increase the production of antimicrobials. The enhanced virulence in the presence of GlcNAc from Gram-positive bacteria was demonstrated *in vivo* in a *Drosophila* and *Galleria mellonella* infection model (Korgaonkar et al., 2013; Whiley et al., 2014). Both GlcNAc and AI-2 sensing are examples where *P. aeruginosa* can sense its environment and generate the appropriate response to eliminate competitors by producing several virulence factors, which also has a negative impact on the host.

## *P. aeruginosa* produces a wide variety of molecules that inhibit *S. aureus in vitro*

*P. aeruginosa* produces many molecules to compete with other microorganisms for space and nutrients. The number of molecules, quantities produced and even the structure of these molecules vary between different strains of *P. aeruginosa* as well as between different growth conditions (planktonic vs. biofilm; the presence of host factors, antibiotics etc.). In order to achieve a better understanding of complex interplay of the different compounds in *in vivo* biofilm-related infections, it is necessary to dissect this complex system into individual subsystems and investigate each compound individually. In the following sections, we discuss the effect of different molecules produced by *P. aeruginosa* on *S. aureus in vitro*. An overview of the extracellular molecules produced by *P. aeruginosa* and their effect on *S. aureus* is shown in Figure 2.

### *Pseudomonas* quinolone signal regulates the production of anti-staphylococcal 4-hydroxy-2-heptylquinoline N-oxide (HQNO)

*P. aeruginosa* strongly reduces or completely outcompetes *S. aureus* during co-culture in many *in vitro* model systems, both planktonic and biofilm (Palmer et al., 2005; Baldan et al., 2014; DeLeon et al., 2014). This anti-staphylococcal activity of *P. aeruginosa* was first described by Lightbown and Jackson (1956), who identified 4-hydroxy-2-heptylquinoline N-oxide (HQNO) as a major compound produced by *P. aeruginosa* that inhibited the cytochrome systems of some bacteria, including *S. aureus* (Lightbown and Jackson, 1956). The same phenomenon was again described by Machan et al. in 1991 by testing the culture supernatant of fifty *P. aeruginosa* clinical isolates on 261 staphylococci (Machan et al., 1991). The growth of all staphylococci was reduced by each of the *P. aeruginosa* strains, although the extent of inhibition was strain-dependent. The factor responsible for this phenomenon was again identified as HQNO (Machan et al., 1992). HQNO is the major compound produced by the *pqsABCDE* operon, which is regulated by the quorum-sensing system PQS. Although HQNO is described as an antistaphylococcal compound, it has no lytic activity against *S. aureus* itself but rather slows down the growth by inhibiting oxidative respiration (Figure 2, right upper panel; Williams and Camara, 2009). Exposure to an HQNO source suppresses the growth of *S. aureus*, resulting in small colonies which are easily missed in diagnostic cultures (Hoffman et al., 2006). These so-called SCVs represent a different phenotype with specific characteristics and will be discussed later in this review. Furthermore, HQNO can be detected at active concentrations in the sputum of CF patients infected with *P. aeruginosa*, suggesting that HQNO has the same effect in the lungs of CF patients as it has *in vitro* (Hoffman et al., 2006). Although HQNO is one of the most important and well-studied antistaphylococcal compounds, it is not the only factor slowing down the growth and inhibiting oxidative respiration in staphylococci.

### Pyocyanin inhibits oxidative respiration in *S. aureus*

Pyocyanin is one of the numerous pigmented phenazines produced by *P. aeruginosa* and an important virulence factor. Pyocyanin is produced during *Pseudomonas* biofilm formation, has a role in acute and chronic airway infections, enables anaerobic survival and serves as a redox-active antimicrobial compound (Biswas et al., 2009; Caldwell et al., 2009). Furthermore, by its inter-and intracellular signaling, pyocyanin enables *P. aeruginosa* to successfully compete with other bacteria and even fungi (Gibson et al., 2009; Toyofuku et al., 2010; Tashiro et al., 2013). Like HQNO, pyocyanin also blocks the oxidative respiration and inhibits growth of *S. aureus*, also selecting for the SCV phenotype (Biswas et al., 2009; Figure 2, right upper panel). The production of pyocyanin can be observed after 8 h of culture, around the same time when a strong reduction of *S. aureus* cells occurs during co-culture (Biswas et al., 2009; Tashiro et al., 2013). Furthermore, the presence of Gram-positive organisms, including some *Staphylococcus* spp., can induce pyocyanin production in *P. aeruginosa* by stimulating the PQS system (Korgaonkar and Whiteley, 2011; Whiley et al., 2014; Figure 2, middle panel). In addition, exposure to pyocyanin in the airways leads to pulmonary damage and contributes to CF pathogenesis (Caldwell et al., 2009). Therefore, pyocyanin seems to be an antagonistic compound secreted to provide a competitive advantage to *P. aeruginosa* by harming *S. aureus*, other Gram-positive bacteria as well as the host.

### LasA protease or staphylolysin effectively lyses *S. aureus* cells

*P. aeruginosa* secretes a staphylolytic endopeptidase called LasA protease or staphylolysin, which degrading pentaglycine in the cell wall of *S. aureus* causing cell lysis (Kessler et al., 1993). *P. aeruginosa* might use LasA protease to compete with staphylococci but also to acquire iron from *S. aureus* (Mashburn et al., 2005; Figure 2, left lower panel). Because freely available iron is often limited, *P. aeruginosa* has developed several strategies to scavenge iron, like the synthesis of iron chelating siderophores, pyoverdin, and pyochelin (Diggle et al., 2007). Transcription patterns of iron-regulated genes of *P. aeruginosa* in the presence of *S. aureus in vivo* are the same as in high-iron conditions *in vitro*, suggesting that *S. aureus* might be an iron source for *P. aeruginosa* (Mashburn et al., 2005). This type of interaction is, however, only important when both species are located close together, like in multi-species biofilms (Mashburn et al., 2005). The specificity of the LasA protease for staphylococci makes it a potential therapeutic candidate against staphylococcal infections especially those cause by antibiotic resistant MRSA strains as shown in a rat model of endophthalmitis (Barequet et al., 2009). However, *S. aureus* can survive LasA by the emergence of L-form-like colonies, which lack a cell wall (Falcon et al., 1989), although the role of L-form like colonies in disease remains rather vague.

### Cis-2-decenoic acid induces biofilm dispersal in a broad range of organisms including *P. aeruginosa*

Interspecies competition in biofilms not only occurs by inhibiting or killing the other species but also by inducing its dispersal. Biofilm dispersal is mainly induced when the environment becomes less favorable, like in case of nutrient depletion, and is extensively reviewed by Petrova and Sauer (2016). The exact mechanisms that induce biofilm dispersal are currently unknown, although several factors have been investigated (Hall-Stoodley and Stoodley, 2005; Davies and Marques, 2009). Since most bacteria reside in a biofilm consisting of multiple species *in vivo*, the dispersal signal must be recognized by a wide range of species (Davies and Marques, 2009). One class of such molecules are the *cis*-monosaturated fatty acids, which are small extracellular messenger molecules with broad inter-phylum and even inter-kingdom activities (Davies and Marques, 2009). *P. aeruginosa* produces *cis*-2-decenoic acid, which induces a dispersion response in biofilms formed by a range of Gram-negative and Gram-positive bacteria, including *S. aureus* (Figure 2, left upper panel), yeast as well as in *P. aeruginosa* (Davies and Marques, 2009). Interestingly, Davies et al showed that dispersion was only induced when the microcolonies reached a minimum of 40 μm diameter and 10 μm of thickness, indicating that a certain threshold concentration is needed for *cis*-2-decenoic acid to become active (Davies and Marques, 2009). This molecule could possibly be employed to disrupt biofilms on surfaces, followed by disinfectants that can successfully clear planktonic bacteria.

### Rhamnolipids promote biofilm dispersal and inhibit adhesion

Most *P. aeruginosa* strains produce rhamnolipids, biosurfactants consisting of one or two rhamnose molecules linked to one or two fatty acids (Soberon-Chavez et al., 2005). While *cis*-2-decenoic acid is mainly used as a common signal for dispersion at the final biofilm stages, rhamnolipids are used to dislodge competing bacteria from the biofilm. Many different rhamnolipid homologs are produced, depending on the *Pseudomonas* strain and carbon source, and their synthesis is quorum-sensing regulated (Soberon-Chavez et al., 2005). Rhamnolipids were shown to reduce the surface tension and to have an anti-adhesive and antimicrobial effect on many micro-organisms (Haba et al., 2003; Rodrigues et al., 2006; Zezzi do Valle Gomes and Nitschke, 2012). The amphiphilic nature of rhamnolipids enables them to intercalate into the cell membranes of different microorganisms and form complexes, thereby permeabilizing the membranes and causing leakage of intracellular material (Sotirova et al., 2008). Gram-positive organisms seem more susceptible to rhamnolipid permeabilization than Gram-negative because the presence of lipopolysaccharides protects the cell membranes of the latter against the effect of surfactants (Soberon-Chavez et al., 2005). Furthermore, rhamnolipids were shown to promote biofilm dispersal in many different microorganisms, including *P. aeruginosa* itself, although this effect is strain dependent (Rodrigues et al., 2006). For *S. aureus* and *S. epidermidis*, rhamnolipids were shown to induce biofilm dispersal and to inhibit adhesion in a dose-dependent manner (Rodrigues et al., 2006; Zezzi do Valle Gomes and Nitschke, 2012; Pihl et al., 2013; Figure 2, left upper panel).

### Long-chain AHLs reduce growth and virulence of *S. aureus*

The N-acylhomoserine lactone (AHL) system is the most important and most extensively studied quorum-sensing system in *P. aeruginosa*. Many diverse AHLs are produced by various Gram-negative bacteria, all consisting of a homoserine lactone ring that is N-acylated with a fatty acyl group (Jimenez et al., 2012). The length of the acyl chains may vary from 4 to 18 carbons and *P. aeruginosa* mainly produces a short-chain C4-HSL and a long-chain 3-oxo-C12-HSL, although other lengths might also occur (Jimenez et al., 2012). The production of many virulence factors is regulated by AHL, including that of pyocyanin and rhamnolipids (Jimenez et al., 2012). Although it is currently not described that Gram-positive bacteria produce AHLs, they might still be influenced by them (Qazi et al., 2006). For example, growth of *S. aureus* is inhibited by several long chain 3-oxo-AHLs (including C8, C10, C12, and C14 chains) in a concentration dependent manner, with C12 and C14 being the most effective (Qazi et al., 2006). At concentrations below growth inhibition, the function of staphylococcal accessory regulator *sarA* and accessory gene regulator *agr* are strongly reduced (see Section SCV Induction by *P. aeruginosa* is Sigma B Dependent), and consequently their dependent virulence factors like hemolysins, TSST-1, protein A, and fibronectin-binding proteins (Figure 2, right lower panel; Qazi et al., 2006). Moreover, inhibition of *agr* might lead to more biofilm formation in *S. aureus* due to reduced detachment, although this study only tested planktonic conditions and requires further research (Qazi et al., 2006). The short chain AHL produced by *P. aeruginosa* seems to have no effect on growth and *agr* expression of *S. aureus* (Qazi et al., 2006). Furthermore, long-chain AHLs produced by other Gram-negative bacteria are likely to have similar effects in *S. aureus*, although, again, more studies are required here.

### *P. aeruginosa* might also cause increased expression of *S. aureus* virulence factors

As described before, wounds infected with both *S. aureus* and *P. aeruginosa* generally show delayed closure compared to the single species infected wounds (Dalton et al., 2011; Seth et al., 2012). In addition to host related factors, one of the reasons for this phenomenon might be the upregulation of *S. aureus* virulence factors during co-infection, as was demonstrated for the MRSA strain USA300 (Pastar et al., 2013). Interestingly, although the growth of USA300 was strongly inhibited by *P. aeruginosa in vitro*, this effect was much weaker *in vivo* (Pastar et al., 2013). Furthermore, co-infection of wounds in a pig model induced *S. aureus* virulence factors *hla* and *pvl*, encoding α-hemolysin and Panton-Valentine leucocidin (Figure 2, right lower panel; Pastar et al., 2013). Another example of increased virulence of *S. aureus* in the presence of *P. aeruginosa* is the induction of staphyloxanthin production observed in a white *S. aureus variant* isolated from a soft tissue wound (Antonic et al., 2013). This strain possessed an intact and functional *crtOPQMN* operon, which is essential for production of the staphyloxanthin pigment, but was unable to induce pigment production on its own. Interestingly, staphyloxanthin production was induced by a *P. aeruginosa* co-isolate (Antonic et al., 2013). Furthermore, the pigment production in a characteristically golden-yellow *S. aureus* strain, which was co-isolated with the *Pseudomonas* strain and the white *S. aureus* variant, remained unaffected. However, this contradicts other studies that report an inhibition of *S. aureus* pigment production by pyocyanin and pyoverdin produced by *P. aeruginosa* (Biswas et al., 2009). Another result of this study (Antonic et al., 2013) that is in contradiction with other studies is that there was an unchanged expression of *sigB* that encodes the alternative transcription factor sigma B and which is previously reported to be upregulated in the presence of *P. aeruginosa* (Mitchell et al., 2010). The discrepancies in results between different studies indicate the importance of co-evolution and adaptation of the different isolates to each other and their environment. Adaptation of *S. aureus* to *P. aeruginosa* might lead to an expression pattern that is similar to a stress-resistant phenotype.

## *S. aureus* survives in the presence of *P. aeruginosa* as the small colony variant phenotype

As a defense mechanism, *S. aureus* has also devised strategies to survive in the presence of *P. aeruginosa*. One of these is the switch to the SCV, a well-characterized phenotype detected in various diseases, including CF and device-related infections (Proctor et al., 2006). SCVs appear as small, smooth colonies on a culture plate and grow significantly slower compared to wild type colonies. The SCV phenotype might appear naturally and is caused by a defective or inhibited electron transport pathway that switches *S. aureus* to a fermentative growth state. In addition to a decreased growth rate, SCVs also demonstrate decreased ATP yield, decreased pigmentation and often hemin or menadione auxotrophy (Proctor et al., 2006; Biswas et al., 2009). Remarkably, the switch to a SCV phenotype increases survival of *S. aureus* in unfavorable conditions as it exhibits an increased aminoglycoside resistance, biofilm formation, and intracellular survival (Hoffman et al., 2006; Proctor et al., 2006; Biswas et al., 2009; Atalla et al., 2011). Prolonged co-culture with *P. aeruginosa* or exposure to pure HQNO leads to a high proportion of stable *S. aureus* SCVs, an effect that is increased by the presence of aminoglycosides (Hoffman et al., 2006). It has also been proposed that the reason why *S. aureus* and *P. aeruginosa* are not frequently detected together in diagnostic cultures of sputum of CF patients is because of the existence of *S. aureus* as SCVs that are more difficult to detect due to their small size and fastidious growth requirements (Proctor et al., 2006; Atalla et al., 2011).

### SCV induction by *P. aeruginosa* is sigma B dependent

After the induction of the SCV phenotype during exposure to HQNO, the expression of three main regulatory mechanisms of virulence and biofilm formation is altered. First, the alternative transcription factor sigma B (SigB) is upregulated (Mitchell et al., 2010). SigB regulates the general stress response of Gram-positive bacteria, repressing the expression of most exoenzymes and toxins, stimulating the expression of adhesins and promoting the persistence of *S. aureus* in host cells (van Schaik and Abee, 2005; Atalla et al., 2011). Second, stimulation of SigB was shown to stimulate the expression of the Staphylococcal accessory regulator SarA, which modulates the expression of the pore-forming toxin α-hemolysin (*hla*) and increases biofilm formation (Valle et al., 2003; Oscarsson et al., 2006; Mitchell et al., 2010). In addition, SigB represses a third important regulator of *S. aureus* biofilm formation, the accessory gene regulator (*agr*) system, which induces biofilm dispersal thereby decreasing the total biomass and increases the expression of *hla* (Boles and Horswill, 2008; Mitchell et al., 2010; Atalla et al., 2011). In conclusion, HQNO reduces the production of the toxin *hla* by increasing the expression of SigB, leading to an increased expression of SarA and a decreased expression of *agr*. Reduced expression of toxins helps *S. aureus* to remain intracellular and thus increase its chances of survival in the human host. The stimulation of sarA by upregulation of SigB might be counteracted by long chain AHLs, which inhibit sarA (Figure 2, right lower panel). The net effect is probably dependent on the *Pseudomonas* strain involved since the production of both factors might be variable between isolates (Qazi et al., 2006; Fugère et al., 2014).

## *P. aeruginosa* and *S. aureus* in dual species biofilms

**Figure 3:**
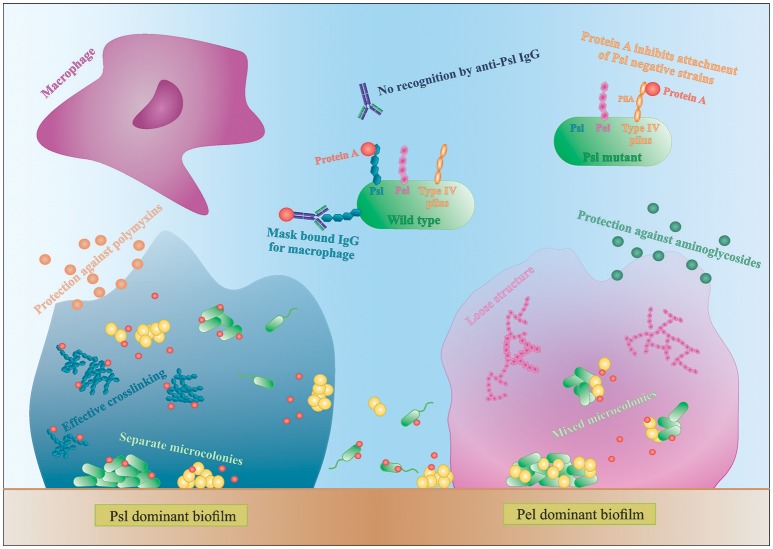
**Interactions in mixed species biofilms**. Differences in biofilm formation by *S. aureus* with a Psl- or Pel-dominant *P. aeruginosa* strain. IgG, immunoglobulin G.

During dual species biofilm formation, the balance of attacking, evading and counter-attacking is even more important and the properties of each strain as well as some environmental factors will determine if a dual species biofilm will be formed. For example, the presence of environmental selection pressure, like antibiotics or the host immune system, stimulates a more synergistic relationship and biofilm formation as the tolerance of *S. aureus* to antibiotics is significantly higher during co-culture with *P. aeruginosa* (DeLeon et al., 2014; Kumar and Ting, 2015). In the following paragraphs we describe how certain extracellular factors influence the structure, characteristics and composition of the dual species biofilms (Figure 3).

### *P. aeruginosa* extracellular polysaccharides are important in the formation of multi-species biofilms

*P. aeruginosa* produces three main exopolysaccharides (EPS): alginate, Pel, and Psl, which form the extracellular matrix in the biofilm and have a structural and protective function (Leid et al., 2005; Ryder et al., 2007; Colvin et al., 2011, 2012). Pel and Psl are the main EPS in non-mucoid strains (Colvin et al., 2012). The Psl polysaccharide is recently identified as repeating units of glucose-, mannose-, and rhamnose-sugars and is mainly produced during the attachment phase of the biofilm (Colvin et al., 2012). Psl-positive strains have an elastic matrix with highly effective cross-linking of the matrix components (Chew et al., 2014). Pel is glucose-rich and is mainly involved in pellicle formation and later stages of biofilm formation (Colvin et al., 2012). Contrary to Psl, Pel dominant strains form loose biofilm structures since Pel reduces the effective cross-linking in the matrix network (Figure 3; Chew et al., 2014). Consequently, Pel-mediated loosening of the *P. aeruginosa* biofilm allows *S. aureus* to infiltrate into the biofilm and form multi-species biofilms (Chew et al., 2014). In contrast, the role of Psl in multi-species biofilm formation is not very clear. Chew et al showed that co-culture of a Psl-positive strain (PAO1) and *S. aureus* resulted in separated microcolonies without much association between both species (Chew et al., 2014). Billings et al., on the other hand, showed that *S. aureus* was incorporated in the air-liquid interface of a Psl producing *P. aeruginosa* biofilm. Both studies use the same biofilm assay and the same *P. aeruginosa* strains, PAO1 and mutants derived from PAO1, but different *S. aureus* strains. Because the biofilm structure and dual species interactions are dependent on both *P. aeruginosa* and the *Staphylococcus* strains, this might explain the discrepancy between the studies. Nonetheless, both studies concluded that the EPS provides protection against antibiotics to all inhabitants of the biofilm, even the non-producers, although the biofilm as a whole is weakened (Billings et al., 2013). More specifically, Psl functions as a protective barrier against the antibiotics colistin and polymyxin B, whereas Pel offers a protective barrier against aminoglycosides (Figure 3; Colvin et al., 2011; Billings et al., 2013). These findings indicate that a minimum amount of EPS per cell present in the biofilm is needed for optimal protection against antibiotics (Billings et al., 2013). These data suggests, if *S. aureus* is able to survive killing by *P. aeruginosa* and to co-exist in a multi-species biofilm, it benefits from the antimicrobial barrier formed by the *P. aeruginosa* matrix components. However, the third EPS, alginate, was not shown to have an effect on *S. aureus* and *S. epidermidis* biofilm formation. Alginate is mainly associated with chronic infections as its overproduction leads to the mucoid phenotype that frequently arises during long-term CF lung infection (Ryder et al., 2007). The switch to a mucoid phenotype contributes to the establishment of chronic colonization since alginate offers structural protection against uptake by macrophages and antimicrobials by forming a barrier limiting the penetration of antimicrobials, macrophages and macrophage-derived products, such as the pro-phagocytic cytokine IFN-γ (Leid et al., 2005; Ryder et al., 2007). Moreover, Leid et al. suggest that alginate might cause the transition from acute to chronic infection by limiting IFN-mediated clearance by macrophages, which is the main mechanism of bacterial clearance during acute infection (Leid et al., 2005).

### *S. aureus* protein a binds to Psl and type IV Pili of *P. aeruginosa*

In addition to EPS, other extracellular factors are important in dual species biofilm formation. Yang et al showed that only *P. aeruginosa* strains producing type IV pili co-aggregate with *S. aureus* in microcolonies (Yang et al., 2011). Type IV pili probably facilitate biofilm formation by binding to extracellular DNA (eDNA), which is derived from dead bacteria and part of the biofilm matrix (Yang et al., 2011). *P. aeruginosa* strains defective for the production of type IV pili or even treating the biofilm with DNAse I was shown to reduce the growth of mixed-species microcolonies (Yang et al., 2011). Furthermore, using a single *S. aureus* laboratory strain, Armbruster et al. showed inhibition of surface attachment of some *P. aeruginosa* clinical isolates due to the secretion of protein A (SpA) by *S. aureus*. SpA is a cell-wall associated extracellular adhesive protein of *S. aureus* that mediates biofilm formation and disrupts phagocytosis (by binding to the Fc portion of IgG antibodies) and its secretion was shown to be increased in artificial sputum (Armbruster et al., 2016). Secreted SpA was shown to specifically bind both Psl and type IV pili of *P. aeruginosa*, stressing the importance of these two molecules in multispecies interactions (Figure 3; Armbruster et al., 2016). In a Psl producing *P. aeruginosa*, all SpA seem to bind to the Psl, leaving the type IV pili free to mediate biofilm formation. In absence of Psl, SpA binds to the PilA component of type IV pili and inhibits adhesion of *P. aeruginosa* (Armbruster et al., 2016). Furthermore, SpA seems to protect *P. aeruginosa* from phagocytosis, as the Psl-SpA complex is no longer recognized by anti-Psl IgG antibodies. SpA can also bind to the Fc domain of anti-Psl IgG antibodies and prevent recognition by neutrophils (Figure 3; Armbruster et al., 2016).

## *P. aeruginosa* and other staphylococci

### *P. aeruginosa* induces biofilm dispersal in *S. epidermidis*

The antistaphylococcal molecules produced by *P. aeruginosa* are also active against other staphylococci, including *S. epidermidis*, although some are more resistant to killing compared to *S. aureus*. *P. aeruginosa* was shown to effectively inhibit and disrupt established *S. epidermidis* biofilms and induce detachment without killing during dual species biofilm formation (Qin et al., 2009; Pihl et al., 2010a,b). After co-inoculation in equal proportions, *P. aeruginosa* and *S. epidermidis* could coexist for up to 18 h. After this time point, the *S. epidermidis* cells in the biofilm are lysed by *P. aeruginosa* (Pihl et al., 2010a). These data suggest that there are two stages in interactions between *P. aeruginosa* and *S. epidermidis*, the first includes the induction of detachment of viable *S. epidermidis* cells from the biofilm, while in the second stage cell lysis causes the total detachment (Pihl et al., 2010a). Similar to *S. aureus*, the effect of *P. aeruginosa* on *S. epidermidis* is strain dependent as some *P. aeruginosa* strains have a more pronounced effect on some *S. epidermidis* strains while others are more resistant to *P. aeruginosa* (Pihl et al., 2010b). Nevertheless, extracellular products that prevent initial attachment of some *S. epidermidis* strains to surfaces might be an interesting option for the development of coatings for indwelling medical devices, like peritoneal dialysis catheters (Pihl et al., 2013). Moreover, in this model, *P. aeruginosa* supernatant components replaced serum proteins on the catheter surface and reduced *S. epidermidis* attachment (Pihl et al., 2013). In addition, exposure of a *S. epidermidis* biofilm on a catheter to *P. aeruginosa* supernatant also caused dispersal of *S. epidermidis* (Pihl et al., 2013). The dispersed cells are, however, not killed making it less suitable as a treatment option and only interesting as a prevention strategy.

### Yayurea A and B from the *S. intermedius* group are quorum-quenching molecules which provide protection against gram negative bacteria

*P. aeruginosa* is originally an environmental bacterium and shares a niche with many other, non-pathogenic staphylococci like the *Staphylococcus intermedius* group consisting of *S. delphini, S. intermedius, S. lutrae, S. pseudointermedius* and *S. schleiferi*. All are common colonizers of various animals and rarely occur in humans (Simou et al., 2005; Ruscher et al., 2009). This group of staphylococci produces two low molecular weight compounds, yayurea A and B, that inhibit the production of quorum-sensing regulated products in Gram negative bacteria and provide protection against extracellular compounds produced by *P. aeruginosa* (Chu et al., 2013). For example, the growth of *S. delphini* is not suppressed by respiratory toxins during co-culture with *P. aeruginosa*. Moreover, *S. delphini* is able to completely inhibit the production of pyocyanin (Chu et al., 2013). The quorum-quenching effect of yayurea A and B covers a broad spectrum of Gram negative bacteria, including *P. aeruginosa, Serratia marcescens, Vibrio harveyi*, and *Chromobacterium subtsugae* (Chu et al., 2013). Quenching of the quorum-sensing system of these Gram negative bacteria does not kill them but rather maintains their physiological state as if the cell density is low, even though density is in fact high. This increases the chances of survival of the staphylococci since toxin production usually begins at high cell density (Chu et al., 2013). Surprisingly, other staphylococci seem to be resistant to both molecules, even though they are not producers (Chu et al., 2013). Interestingly, *S. aureus* is protected from killing by *P. aeruginosa* when yayurea A and B are added to the medium without the former having to undergo physiological changes (SCV formation), amd represent promising candidates for inhibition studies of P. aeruginosa virulence and biofilm formation.

### Variations in CydAB from *S. carnosus* provides protection against killing by *P. aeruginosa*

In addition to the *S. intermedius* group, several other non-pathogenic staphylococci (*S. carnosus, S. piscifermentans*, and *S. simulans*) seem to be resistant to respiratory toxins secreted by *P. aeruginosa* due to alterations in the cydAB genes. These genes encode the two subunits of cytochrome bd quinol oxidase, of which homologs are also present in the genomes of *S. aureus* and *S. epidermidis* (Voggu et al., 2006). However, only the cytochrome bd quinol oxidase of the first group is resistant to the respiratory toxin, pyocyanin (Voggu et al., 2006). Furthermore, cloning of the *S. carnosus* cydAB cluster into *S. aureus* confers resistance to respiratory inhibitors produced by *P. aeruginosa* (Voggu et al., 2006). Further research showed that, whereas the CydA subunit is more conserved in staphylococci, CydB underwent a microevolution with relatively higher identity within than between the groups of pathogenic and non-pathogenic staphylococci (Voggu et al., 2006). This asymmetric evolution of CydB could be explained by the fact that the non-pathogenic staphylococci frequently inhabit the same environment as *Pseudomonas* spp. and were therefore selected for a higher resistance to respiratory toxins.

## Concluding remarks

Both *P. aeruginosa* and staphylococci are highly versatile organisms, which readily adapt to a wide variety of environments and stress factors. In the first glance, these bacteria seem to have an antagonistic relationship as *P. aeruginosa* produces a wide variety of molecules inhibiting staphylococci and frequently outcompetes *S. aureus* and *S. epidermidis* during co-culture. This antagonistic behavior is mainly shown during planktonic growth and under traditional culture conditions, where no host factors or antibiotics are present. However, under some *in vitro* and *in vivo* circumstances, both bacteria are able to co-exist and form dual species biofilms. These circumstances are dependent on a combination of strain-dependent properties of both species and the presence or absence of certain environmental factors like antibiotics or host factors, the sum of which might tip the balance toward either killing or co-existence. The presence of some sort of selection pressure or presence of a preformed matrix seems to favor dual species biofilm formation whereas planktonic co-culture without selection pressure leads to domination of *P. aeruginosa*. Interspecies competition often leads to an increased production of virulence factors in both *P. aeruginosa* and *S. aureus*, which are also harmful to the human host. In addition, escaping the antistaphylococcal compounds results in a more stress-resistant phenotype of *S. aureus*, which is more difficult to be cleared by the immune system, to be eradicated by antibiotics and to be detected in diagnostic cultures. Furthermore, the presence of an extracellular matrix was shown to be beneficial for all biofilm inhabitants, providing protection against classical antibiotics and the host immune system, although the exact composition might be variable depending on the species present and the environment. The role of these matrix components (exopolysaccharides, eDNA, matrix proteins, host-derived factors etc.) in interspecies interactions and their role in disease pathogenesis provides an exciting opportunity for future research toward better patient care. When strains are co-existing for a longer time, they might evolve to a phenotype that is better adapted to the presence of the other. For example, non-pathogenic staphylococci that are frequently encountering *P. aeruginosa* have developed strategies to continue growing in the presence of *P. aeruginosa* antistaphylcoccal compounds, indicating parallel evolution. Moreover, *S. aureus* and *P. aeruginosa* strains isolated from the same chronic CF lung infection are less sensitive to, and produce less, HQNO, respectively. This strain adaptation and the underestimation of the co-existence of *P. aeruginosa* and *S. aureus* might still have a large impact on the clinical outcome of a patient and therefore should be a subject of continuing investigation.

## Author contributions

AH collected literature. All authors contributed in drafting the review.

## Funding

AH was supported by funding from the Innovative Medicines Initiative project COMBACTE-MAGNET (Combatting Bacterial Resistance in Europe—Molecules Against Gram Negative Infections) (IMI Grant Agreement No 115737-2) and funding from Research Foundation Flanders (FWO-F, G.0513.12).

### Conflict of interest statement

The authors declare that the research was conducted in the absence of any commercial or financial relationships that could be construed as a potential conflict of interest.
